# Supplementary material for: Hot spot prediction in protein-protein interactions by an ensemble system
Source: BMC Syst Biol. 2018 Dec 31;12(Suppl 9):132. doi: 10.1186/s12918-018-0665-8 (PMC6311905; doi:10.1186/s12918-018-0665-8)
Supplement: Supplementary file 2 — All relevant MATLAB code. A simple Matlab implement of our predictor. (ZIP 19,742 kb) [file 12918_2018_665_MOESM2_ESM.zip › Additional file 2/Read ASA/ASA website/dbMPIKT/1T09A.html]

 NetSurfP - Protein Surface Accessibility and Secondary Structure Predictions

  

|  |  |  |
| --- | --- | --- |
|  |  | NetSurfP - Protein Surface Accessibility and Secondary Structure Predictions  Technical University of Denmark |

  


---

```
# For publication of results, please cite:
# A generic method for assignment of reliability scores applied to solvent accessibility predictions.
# Bent Petersen, Thomas Nordahl Petersen, Pernille Andersen, Morten Nielsen and Claus Lundegaard
# BMC Structural Biology 2009, 9:51 doi:10.1186/1472-6807-9-51
#
# Column 1: Class assignment - B for buried or E for Exposed - Threshold: 25% exposure, but not based on RSA
# Column 2: Amino acid
# Column 3: Sequence name
# Column 4: Amino acid number
# Column 5: Relative Surface Accessibility - RSA
# Column 6: Absolute Surface Accessibility
# Column 7: Z-fit score for RSA prediction
# Column 8: Probability for Alpha-Helix
# Column 9: Probability for Beta-strand
# Column 10: Probability for Coil
E M  1T09A                  1    0.670 134.027  -1.715   0.003   0.003   0.994
E S  1T09A                  2    0.446  52.318  -1.183   0.020   0.205   0.775
E K  1T09A                  3    0.569 116.940   0.555   0.022   0.359   0.619
E K  1T09A                  4    0.474  97.440   0.037   0.022   0.359   0.619
E I  1T09A                  5    0.467  86.302  -0.461   0.021   0.279   0.699
E S  1T09A                  6    0.497  58.260  -0.298   0.019   0.141   0.840
E G  1T09A                  7    0.663  52.162   1.006   0.004   0.138   0.858
B G  1T09A                  8    0.171  13.458   1.331   0.005   0.262   0.733
B S  1T09A                  9    0.226  26.464   1.528   0.004   0.420   0.576
B V  1T09A                 10    0.018   2.828   1.563   0.001   0.900   0.099
B V  1T09A                 11    0.020   3.028   0.956   0.001   0.959   0.040
B E  1T09A                 12    0.037   6.429   0.332   0.001   0.959   0.040
B M  1T09A                 13    0.019   3.762   1.567   0.001   0.900   0.099
B Q  1T09A                 14    0.187  33.452   0.343   0.004   0.420   0.576
B G  1T09A                 15    0.071   5.588  -1.900   0.005   0.045   0.951
B D  1T09A                 16    0.159  22.883  -0.895   0.018   0.019   0.964
B E  1T09A                 17    0.136  23.847  -1.956   0.058   0.017   0.925
B M  1T09A                 18    0.055  11.026  -0.075   0.113   0.043   0.844
B T  1T09A                 19    0.041   5.701   0.277   0.257   0.016   0.727
B R  1T09A                 20    0.229  52.395   0.805   0.923   0.002   0.076
B I  1T09A                 21    0.144  26.658   0.173   0.923   0.002   0.076
B I  1T09A                 22    0.020   3.756   1.418   0.970   0.001   0.030
B W  1T09A                 23    0.046  10.991   1.237   0.970   0.001   0.030
E E  1T09A                 24    0.439  76.641   2.058   0.988   0.000   0.012
B L  1T09A                 25    0.086  15.673   0.748   0.988   0.000   0.012
B I  1T09A                 26    0.013   2.479   1.505   0.988   0.000   0.012
B K  1T09A                 27    0.176  36.121   1.400   0.988   0.000   0.012
E E  1T09A                 28    0.506  88.328   2.153   0.988   0.000   0.012
B K  1T09A                 29    0.038   7.919   0.892   0.970   0.001   0.030
B L  1T09A                 30    0.038   6.903   0.416   0.970   0.001   0.030
E I  1T09A                 31    0.528  97.717   1.745   0.923   0.002   0.076
E F  1T09A                 32    0.382  76.768   0.236   0.782   0.003   0.216
B P  1T09A                 33    0.213  30.239  -0.158   0.246   0.004   0.750
E Y  1T09A                 34    0.718 153.458  -0.585   0.016   0.005   0.979
B V  1T09A                 35    0.106  16.338   1.013   0.005   0.045   0.951
E E  1T09A                 36    0.654 114.201   1.432   0.004   0.138   0.858
B L  1T09A                 37    0.051   9.411   1.116   0.004   0.616   0.381
E D  1T09A                 38    0.467  67.251   1.721   0.001   0.900   0.099
B L  1T09A                 39    0.114  20.928   1.288   0.001   0.959   0.040
E H  1T09A                 40    0.426  77.489   1.236   0.001   0.959   0.040
E S  1T09A                 41    0.460  53.877   0.397   0.001   0.959   0.040
B Y  1T09A                 42    0.110  23.507   1.251   0.001   0.959   0.040
E D  1T09A                 43    0.434  62.511  -0.117   0.001   0.900   0.099
B L  1T09A                 44    0.052   9.466   0.028   0.022   0.552   0.426
B G  1T09A                 45    0.186  14.654  -0.991   0.056   0.142   0.802
B I  1T09A                 46    0.239  44.289  -0.024   0.268   0.043   0.689
E E  1T09A                 47    0.563  98.374   0.742   0.455   0.046   0.498
B N  1T09A                 48    0.088  12.869  -0.319   0.605   0.105   0.290
B R  1T09A                 49    0.152  34.739   0.416   0.701   0.107   0.192
E D  1T09A                 50    0.512  73.794  -0.034   0.660   0.049   0.291
E A  1T09A                 51    0.628  69.206   0.574   0.522   0.016   0.462
E T  1T09A                 52    0.424  58.767  -0.744   0.321   0.003   0.675
E N  1T09A                 53    0.575  84.121  -1.501   0.053   0.005   0.942
E D  1T09A                 54    0.543  78.275  -0.282   0.018   0.019   0.964
E Q  1T09A                 55    0.361  64.421   0.137   0.018   0.019   0.964
B V  1T09A                 56    0.059   9.084   0.501   0.005   0.015   0.979
B T  1T09A                 57    0.135  18.724   0.910   0.246   0.004   0.750
E K  1T09A                 58    0.636 130.763   0.728   0.923   0.002   0.076
E D  1T09A                 59    0.488  70.364   0.872   0.923   0.002   0.076
B A  1T09A                 60    0.020   2.171   0.861   0.970   0.001   0.030
B A  1T09A                 61    0.094  10.370   0.847   0.970   0.001   0.030
E E  1T09A                 62    0.530  92.678   1.889   0.970   0.001   0.030
B A  1T09A                 63    0.115  12.706   1.055   0.970   0.001   0.030
B I  1T09A                 64    0.014   2.516   1.143   0.970   0.001   0.030
E K  1T09A                 65    0.397  81.601   1.707   0.938   0.007   0.055
E K  1T09A                 66    0.614 126.300   1.496   0.879   0.010   0.111
B H  1T09A                 67    0.043   7.804   0.604   0.406   0.004   0.590
E N  1T09A                 68    0.258  37.742   0.446   0.053   0.043   0.903
B V  1T09A                 69    0.019   2.936   0.864   0.021   0.451   0.528
B G  1T09A                 70    0.021   1.684   0.636   0.011   0.918   0.071
B V  1T09A                 71    0.015   2.382   1.296   0.011   0.918   0.071
B K  1T09A                 72    0.039   8.022   0.252   0.011   0.918   0.071
B C  1T09A                 73    0.018   2.541   0.901   0.022   0.552   0.426
B A  1T09A                 74    0.045   4.959  -0.637   0.020   0.205   0.775
B T  1T09A                 75    0.047   6.533  -0.939   0.020   0.205   0.775
B I  1T09A                 76    0.135  24.938  -1.136   0.019   0.141   0.840
B T  1T09A                 77    0.133  18.405  -0.259   0.018   0.047   0.935
B P  1T09A                 78    0.222  31.516   0.484   0.058   0.017   0.925
E D  1T09A                 79    0.556  80.091  -0.185   0.115   0.016   0.868
E E  1T09A                 80    0.334  58.420  -0.555   0.181   0.016   0.803
E K  1T09A                 81    0.521 107.108  -1.388   0.181   0.016   0.803
E R  1T09A                 82    0.433  99.271  -0.685   0.181   0.016   0.803
B V  1T09A                 83    0.206  31.708  -1.128   0.181   0.016   0.803
E E  1T09A                 84    0.546  95.316  -0.752   0.257   0.016   0.727
E E  1T09A                 85    0.576 100.697  -1.407   0.257   0.016   0.727
E F  1T09A                 86    0.377  75.724  -1.184   0.181   0.016   0.803
E K  1T09A                 87    0.530 109.042  -1.732   0.181   0.016   0.803
B L  1T09A                 88    0.207  37.920  -0.681   0.113   0.043   0.844
E K  1T09A                 89    0.387  79.668  -1.018   0.113   0.043   0.844
E Q  1T09A                 90    0.601 107.267  -1.890   0.113   0.043   0.844
E M  1T09A                 91    0.589 117.859  -2.363   0.053   0.043   0.903
B W  1T09A                 92    0.249  59.957  -1.063   0.052   0.084   0.864
E K  1T09A                 93    0.294  60.558  -0.824   0.113   0.087   0.800
B S  1T09A                 94    0.188  22.010  -2.047   0.113   0.043   0.844
B P  1T09A                 95    0.195  27.628  -1.756   0.622   0.015   0.363
B N  1T09A                 96    0.189  27.640  -0.775   0.802   0.014   0.185
B G  1T09A                 97    0.180  14.135  -0.475   0.879   0.010   0.111
E T  1T09A                 98    0.258  35.840   0.522   0.938   0.007   0.055
B I  1T09A                 99    0.029   5.328   0.157   0.938   0.007   0.055
B R  1T09A                100    0.163  37.327   0.521   0.938   0.007   0.055
E N  1T09A                101    0.481  70.389   0.366   0.879   0.010   0.111
E I  1T09A                102    0.334  61.827   0.606   0.802   0.014   0.185
B L  1T09A                103    0.101  18.493   0.339   0.430   0.016   0.555
E G  1T09A                104    0.593  46.661   0.070   0.058   0.017   0.925
B G  1T09A                105    0.167  13.119  -0.220   0.018   0.088   0.893
B T  1T09A                106    0.137  18.974   0.433   0.022   0.552   0.426
B V  1T09A                107    0.017   2.674   1.351   0.002   0.816   0.182
B F  1T09A                108    0.036   7.125  -0.227   0.018   0.846   0.136
B R  1T09A                109    0.027   6.091   0.757   0.003   0.718   0.279
B E  1T09A                110    0.050   8.665  -0.179   0.004   0.616   0.381
B A  1T09A                111    0.032   3.526   0.071   0.004   0.514   0.481
B I  1T09A                112    0.056  10.397   0.399   0.021   0.756   0.223
B I  1T09A                113    0.125  23.125  -0.127   0.021   0.756   0.223
B C  1T09A                114    0.019   2.682   0.564   0.022   0.552   0.426
E K  1T09A                115    0.275  56.650   0.217   0.021   0.451   0.528
B N  1T09A                116    0.272  39.865  -0.974   0.005   0.262   0.733
B I  1T09A                117    0.105  19.499   0.289   0.018   0.088   0.893
E P  1T09A                118    0.459  65.175  -1.003   0.018   0.019   0.964
E R  1T09A                119    0.410  93.982  -1.446   0.058   0.017   0.925
B L  1T09A                120    0.128  23.418  -0.351   0.053   0.043   0.903
E V  1T09A                121    0.459  70.610  -0.594   0.052   0.084   0.864
E S  1T09A                122    0.463  54.287  -0.904   0.053   0.043   0.903
E G  1T09A                123    0.466  36.674  -1.688   0.053   0.043   0.903
B W  1T09A                124    0.256  61.664  -0.733   0.053   0.043   0.903
E V  1T09A                125    0.539  82.783  -1.053   0.053   0.043   0.903
E K  1T09A                126    0.377  77.652  -0.781   0.053   0.043   0.903
B P  1T09A                127    0.242  34.397  -0.942   0.113   0.087   0.800
B I  1T09A                128    0.086  15.966  -0.359   0.069   0.386   0.545
B I  1T09A                129    0.119  22.052  -0.571   0.079   0.592   0.329
B I  1T09A                130    0.133  24.531  -0.521   0.087   0.683   0.230
B G  1T09A                131    0.134  10.569  -0.104   0.087   0.683   0.230
B R  1T09A                132    0.228  52.120  -0.256   0.074   0.484   0.442
E H  1T09A                133    0.419  76.180  -0.688   0.135   0.317   0.548
E A  1T09A                134    0.370  40.730  -0.547   0.118   0.150   0.732
E Y  1T09A                135    0.429  91.677  -0.613   0.118   0.150   0.732
E G  1T09A                136    0.564  44.363  -1.196   0.052   0.084   0.864
E D  1T09A                137    0.550  79.269  -0.816   0.052   0.084   0.864
E Q  1T09A                138    0.380  67.868  -0.352   0.066   0.296   0.638
B Y  1T09A                139    0.156  33.401  -0.611   0.021   0.451   0.528
E R  1T09A                140    0.414  94.783  -0.046   0.022   0.359   0.619
E A  1T09A                141    0.360  39.650  -1.907   0.004   0.197   0.799
B T  1T09A                142    0.229  31.762  -0.093   0.004   0.420   0.576
B D  1T09A                143    0.194  27.999  -1.822   0.004   0.616   0.381
B F  1T09A                144    0.151  30.326   0.746   0.001   0.900   0.099
E V  1T09A                145    0.363  55.732   0.163   0.001   0.959   0.040
B V  1T09A                146    0.117  18.060  -0.349   0.001   0.900   0.099
E P  1T09A                147    0.374  53.142  -0.663   0.003   0.718   0.279
E G  1T09A                148    0.449  35.328  -0.715   0.005   0.262   0.733
E P  1T09A                149    0.735 104.325  -0.361   0.004   0.085   0.910
E G  1T09A                150    0.444  34.927  -1.256   0.004   0.138   0.858
E K  1T09A                151    0.301  61.998  -0.220   0.004   0.420   0.576
B V  1T09A                152    0.239  36.688  -0.775   0.003   0.718   0.279
B E  1T09A                153    0.159  27.865  -0.842   0.001   0.900   0.099
B I  1T09A                154    0.073  13.542  -0.673   0.001   0.959   0.040
B T  1T09A                155    0.153  21.166   0.474   0.001   0.959   0.040
B Y  1T09A                156    0.092  19.682  -0.554   0.001   0.959   0.040
B T  1T09A                157    0.150  20.833  -0.348   0.002   0.816   0.182
B P  1T09A                158    0.248  35.262  -1.033   0.004   0.514   0.481
E S  1T09A                159    0.605  70.953  -1.184   0.019   0.141   0.840
E D  1T09A                160    0.615  88.622  -1.694   0.053   0.043   0.903
E G  1T09A                161    0.440  34.659  -1.862   0.052   0.084   0.864
E T  1T09A                162    0.471  65.328  -1.589   0.064   0.216   0.721
E Q  1T09A                163    0.465  83.085  -0.719   0.069   0.386   0.545
E K  1T09A                164    0.510 104.866  -0.683   0.022   0.552   0.426
E V  1T09A                165    0.375  57.699  -0.711   0.023   0.655   0.322
E T  1T09A                166    0.481  66.645  -0.796   0.023   0.655   0.322
E Y  1T09A                167    0.397  84.924  -0.112   0.021   0.756   0.223
E L  1T09A                168    0.372  68.168  -0.274   0.021   0.756   0.223
B V  1T09A                169    0.234  35.966  -0.804   0.023   0.655   0.322
B H  1T09A                170    0.279  50.768  -0.677   0.022   0.552   0.426
E N  1T09A                171    0.593  86.830  -1.213   0.064   0.216   0.721
B F  1T09A                172    0.288  57.741  -1.829   0.052   0.084   0.864
E E  1T09A                173    0.485  84.817  -1.416   0.052   0.084   0.864
E E  1T09A                174    0.509  88.975  -1.455   0.052   0.084   0.864
E G  1T09A                175    0.404  31.787  -1.172   0.019   0.141   0.840
E G  1T09A                176    0.287  22.587  -1.122   0.021   0.279   0.699
B G  1T09A                177    0.090   7.067  -0.804   0.023   0.655   0.322
B V  1T09A                178    0.048   7.454   0.007   0.011   0.918   0.071
B A  1T09A                179    0.162  17.885  -0.865   0.011   0.918   0.071
B M  1T09A                180    0.107  21.411  -0.963   0.018   0.846   0.136
B G  1T09A                181    0.081   6.383  -0.821   0.085   0.775   0.140
B M  1T09A                182    0.107  21.471   0.196   0.087   0.683   0.230
B Y  1T09A                183    0.188  40.282   0.436   0.087   0.683   0.230
B N  1T09A                184    0.080  11.741   0.099   0.079   0.592   0.329
B Q  1T09A                185    0.067  12.002  -0.331   0.135   0.317   0.548
E D  1T09A                186    0.322  46.400   0.535   0.938   0.007   0.055
E K  1T09A                187    0.460  94.540   0.667   0.970   0.001   0.030
B S  1T09A                188    0.087  10.232   0.603   0.970   0.001   0.030
B I  1T09A                189    0.014   2.627   1.125   0.970   0.001   0.030
E E  1T09A                190    0.267  46.610   1.272   0.988   0.000   0.012
E D  1T09A                191    0.230  33.114   1.267   0.988   0.000   0.012
B F  1T09A                192    0.022   4.335   1.373   0.988   0.000   0.012
B A  1T09A                193    0.017   1.917   1.403   0.988   0.000   0.012
E H  1T09A                194    0.276  50.204   1.497   0.988   0.000   0.012
B S  1T09A                195    0.128  15.002   1.088   0.988   0.000   0.012
B S  1T09A                196    0.016   1.899   1.101   0.988   0.000   0.012
B F  1T09A                197    0.057  11.360   0.652   0.988   0.000   0.012
E Q  1T09A                198    0.424  75.691   1.769   0.970   0.001   0.030
B M  1T09A                199    0.108  21.671   0.987   0.970   0.001   0.030
B A  1T09A                200    0.018   2.028   0.335   0.970   0.001   0.030
E L  1T09A                201    0.378  69.285   1.197   0.923   0.002   0.076
E S  1T09A                202    0.686  80.411   0.839   0.782   0.003   0.216
E K  1T09A                203    0.474  97.481   0.197   0.246   0.004   0.750
E G  1T09A                204    0.699  55.011  -0.069   0.003   0.003   0.994
B W  1T09A                205    0.204  48.966   0.499   0.005   0.015   0.979
E P  1T09A                206    0.349  49.537   1.521   0.005   0.262   0.733
B L  1T09A                207    0.019   3.424   1.231   0.002   0.816   0.182
B Y  1T09A                208    0.041   8.804   0.349   0.001   0.959   0.040
B L  1T09A                209    0.019   3.552   0.605   0.001   0.959   0.040
B S  1T09A                210    0.018   2.156   0.830   0.001   0.900   0.099
B T  1T09A                211    0.064   8.849   0.006   0.004   0.514   0.481
B K  1T09A                212    0.090  18.492   0.082   0.113   0.043   0.844
B N  1T09A                213    0.179  26.206  -1.038   0.176   0.004   0.820
B T  1T09A                214    0.152  21.110  -0.514   0.502   0.002   0.495
B I  1T09A                215    0.024   4.496  -0.193   0.782   0.003   0.216
B L  1T09A                216    0.052   9.503  -0.119   0.717   0.014   0.269
E K  1T09A                217    0.306  62.924   0.962   0.802   0.014   0.185
B K  1T09A                218    0.157  32.254  -0.416   0.879   0.010   0.111
B Y  1T09A                219    0.038   8.227  -0.930   0.782   0.003   0.216
B D  1T09A                220    0.083  11.931  -0.944   0.923   0.002   0.076
E G  1T09A                221    0.310  24.405  -0.249   0.970   0.001   0.030
B R  1T09A                222    0.088  20.129   0.640   0.988   0.000   0.012
B F  1T09A                223    0.018   3.593   0.956   0.988   0.000   0.012
B K  1T09A                224    0.210  43.238   1.311   0.988   0.000   0.012
E D  1T09A                225    0.436  62.799   1.248   0.988   0.000   0.012
B I  1T09A                226    0.049   9.065   1.027   0.988   0.000   0.012
B F  1T09A                227    0.016   3.191   1.447   0.988   0.000   0.012
E Q  1T09A                228    0.389  69.386   2.133   0.988   0.000   0.012
E E  1T09A                229    0.446  77.846   1.825   0.988   0.000   0.012
B I  1T09A                230    0.038   7.030   0.196   0.970   0.001   0.030
B Y  1T09A                231    0.061  12.993   0.684   0.923   0.002   0.076
E D  1T09A                232    0.568  81.820   1.787   0.858   0.002   0.139
E K  1T09A                233    0.650 133.746   1.444   0.694   0.003   0.303
B Q  1T09A                234    0.188  33.505   0.333   0.406   0.004   0.590
B Y  1T09A                235    0.166  35.581   0.346   0.176   0.004   0.820
E K  1T09A                236    0.700 143.928   1.168   0.115   0.016   0.868
E S  1T09A                237    0.411  48.134  -0.313   0.184   0.043   0.773
E Q  1T09A                238    0.501  89.550   0.333   0.191   0.086   0.723
B F  1T09A                239    0.222  44.515  -0.580   0.199   0.152   0.649
E E  1T09A                240    0.541  94.583  -0.688   0.199   0.152   0.649
E A  1T09A                241    0.564  62.153  -0.715   0.191   0.086   0.723
E Q  1T09A                242    0.394  70.297  -0.700   0.113   0.087   0.800
E K  1T09A                243    0.497 102.151  -0.126   0.052   0.084   0.864
B I  1T09A                244    0.083  15.373  -0.049   0.135   0.317   0.548
E W  1T09A                245    0.360  86.532   0.642   0.268   0.505   0.227
B Y  1T09A                246    0.144  30.858   1.060   0.387   0.545   0.068
B E  1T09A                247    0.244  42.697  -0.028   0.487   0.370   0.144
B H  1T09A                248    0.177  32.196  -0.006   0.487   0.370   0.144
B R  1T09A                249    0.189  43.189   0.935   0.594   0.285   0.121
B L  1T09A                250    0.058  10.675   0.891   0.779   0.100   0.120
B I  1T09A                251    0.021   3.903   1.417   0.938   0.007   0.055
B D  1T09A                252    0.054   7.796  -0.271   0.975   0.003   0.022
B D  1T09A                253    0.088  12.681   0.294   0.975   0.003   0.022
B M  1T09A                254    0.028   5.583   0.709   0.988   0.000   0.012
B V  1T09A                255    0.021   3.258   0.999   0.970   0.001   0.030
B A  1T09A                256    0.069   7.593   0.280   0.970   0.001   0.030
B Q  1T09A                257    0.211  37.667   1.326   0.970   0.001   0.030
B A  1T09A                258    0.021   2.281   1.052   0.923   0.002   0.076
B M  1T09A                259    0.044   8.704  -0.578   0.694   0.003   0.303
E K  1T09A                260    0.319  65.577   0.835   0.406   0.004   0.590
E S  1T09A                261    0.268  31.398   0.945   0.053   0.005   0.942
E E  1T09A                262    0.371  64.866  -0.350   0.016   0.005   0.979
E G  1T09A                263    0.540  42.498   0.027   0.003   0.003   0.994
B G  1T09A                264    0.134  10.562  -0.102   0.018   0.019   0.964
B F  1T09A                265    0.033   6.543   0.248   0.022   0.359   0.619
B I  1T09A                266    0.018   3.312   1.250   0.011   0.918   0.071
B W  1T09A                267    0.018   4.257   1.627   0.006   0.962   0.032
B A  1T09A                268    0.017   1.851   1.371   0.011   0.918   0.071
B C  1T09A                269    0.016   2.232   1.527   0.023   0.655   0.322
B K  1T09A                270    0.102  20.961  -0.264   0.066   0.296   0.638
B N  1T09A                271    0.069  10.058  -1.022   0.184   0.043   0.773
B Y  1T09A                272    0.093  19.831  -0.068   0.339   0.016   0.645
B D  1T09A                273    0.115  16.629  -1.752   0.430   0.016   0.555
B G  1T09A                274    0.064   5.013  -1.536   0.717   0.014   0.269
B D  1T09A                275    0.073  10.534  -0.917   0.802   0.014   0.185
B V  1T09A                276    0.042   6.440   0.582   0.879   0.010   0.111
B Q  1T09A                277    0.026   4.572   0.787   0.938   0.007   0.055
B S  1T09A                278    0.040   4.676   0.273   0.923   0.002   0.076
B D  1T09A                279    0.070  10.159  -0.809   0.923   0.002   0.076
B S  1T09A                280    0.053   6.247   0.373   0.923   0.002   0.076
B V  1T09A                281    0.030   4.596  -0.284   0.923   0.002   0.076
B A  1T09A                282    0.046   5.080  -1.326   0.858   0.002   0.139
B Q  1T09A                283    0.089  15.931  -0.978   0.858   0.002   0.139
B G  1T09A                284    0.090   7.075  -0.588   0.802   0.014   0.185
B Y  1T09A                285    0.074  15.878  -1.199   0.622   0.015   0.363
B G  1T09A                286    0.103   8.122  -1.814   0.257   0.016   0.727
B S  1T09A                287    0.077   9.036  -2.102   0.257   0.016   0.727
B L  1T09A                288    0.061  11.187  -0.563   0.268   0.043   0.689
B G  1T09A                289    0.084   6.650  -2.245   0.191   0.086   0.723
B M  1T09A                290    0.053  10.665  -0.529   0.118   0.150   0.732
B M  1T09A                291    0.027   5.323   0.139   0.125   0.227   0.648
B T  1T09A                292    0.071   9.820  -1.142   0.125   0.227   0.648
B S  1T09A                293    0.037   4.301  -2.592   0.066   0.296   0.638
B V  1T09A                294    0.065  10.037  -1.348   0.069   0.386   0.545
B L  1T09A                295    0.062  11.279  -2.201   0.069   0.386   0.545
B V  1T09A                296    0.058   8.930  -0.050   0.021   0.279   0.699
B C  1T09A                297    0.068   9.505  -1.477   0.018   0.088   0.893
E P  1T09A                298    0.533  75.619  -0.790   0.018   0.019   0.964
E D  1T09A                299    0.683  98.449  -1.300   0.018   0.019   0.964
E G  1T09A                300    0.330  25.955  -0.920   0.018   0.019   0.964
B K  1T09A                301    0.229  47.105  -0.452   0.019   0.141   0.840
B T  1T09A                302    0.124  17.199   0.732   0.023   0.655   0.322
B V  1T09A                303    0.020   3.120   0.597   0.018   0.846   0.136
B E  1T09A                304    0.034   5.957  -0.301   0.018   0.846   0.136
B A  1T09A                305    0.028   3.130  -0.219   0.018   0.846   0.136
B E  1T09A                306    0.063  11.006  -0.092   0.023   0.655   0.322
B A  1T09A                307    0.052   5.686  -0.476   0.021   0.279   0.699
B A  1T09A                308    0.086   9.521  -1.328   0.020   0.205   0.775
B H  1T09A                309    0.140  25.539  -0.713   0.064   0.216   0.721
B G  1T09A                310    0.107   8.421  -1.717   0.064   0.216   0.721
B T  1T09A                311    0.166  23.024  -1.585   0.056   0.142   0.802
B V  1T09A                312    0.059   9.038  -0.395   0.113   0.087   0.800
B T  1T09A                313    0.168  23.371  -0.317   0.113   0.043   0.844
E R  1T09A                314    0.319  73.143  -0.432   0.184   0.043   0.773
B H  1T09A                315    0.305  55.443  -1.097   0.268   0.043   0.689
E Y  1T09A                316    0.308  65.713  -0.715   0.354   0.048   0.598
B R  1T09A                317    0.235  53.792  -1.154   0.455   0.046   0.498
E M  1T09A                318    0.295  59.130  -0.951   0.561   0.047   0.393
E Y  1T09A                319    0.400  85.565  -0.944   0.561   0.047   0.393
E Q  1T09A                320    0.286  51.044  -1.209   0.660   0.049   0.291
E K  1T09A                321    0.281  57.699  -0.395   0.522   0.016   0.462
B G  1T09A                322    0.328  25.821  -1.241   0.339   0.016   0.645
E Q  1T09A                323    0.525  93.783  -0.778   0.181   0.016   0.803
E E  1T09A                324    0.547  95.543  -0.378   0.115   0.016   0.868
B T  1T09A                325    0.285  39.571  -1.165   0.053   0.043   0.903
B S  1T09A                326    0.155  18.166  -0.428   0.052   0.084   0.864
B T  1T09A                327    0.032   4.411  -1.312   0.113   0.087   0.800
B N  1T09A                328    0.059   8.667   0.499   0.053   0.043   0.903
B P  1T09A                329    0.038   5.406  -0.485   0.522   0.016   0.462
B I  1T09A                330    0.064  11.748  -0.412   0.923   0.002   0.076
B A  1T09A                331    0.028   3.108  -0.490   0.970   0.001   0.030
B S  1T09A                332    0.028   3.328   0.325   0.970   0.001   0.030
B I  1T09A                333    0.021   3.940   0.868   0.970   0.001   0.030
B F  1T09A                334    0.075  15.073   0.531   0.970   0.001   0.030
B A  1T09A                335    0.028   3.064  -0.225   0.970   0.001   0.030
B W  1T09A                336    0.034   8.105  -0.700   0.970   0.001   0.030
B T  1T09A                337    0.035   4.813  -0.939   0.970   0.001   0.030
B R  1T09A                338    0.086  19.786  -0.142   0.970   0.001   0.030
B G  1T09A                339    0.019   1.480   0.897   0.970   0.001   0.030
B L  1T09A                340    0.024   4.394   0.704   0.970   0.001   0.030
B A  1T09A                341    0.213  23.517   0.813   0.970   0.001   0.030
B H  1T09A                342    0.128  23.229   0.154   0.923   0.002   0.076
B R  1T09A                343    0.055  12.526   0.361   0.782   0.003   0.216
B A  1T09A                344    0.152  16.717  -1.118   0.502   0.002   0.495
E K  1T09A                345    0.492 101.204   0.235   0.321   0.003   0.675
E L  1T09A                346    0.331  60.679  -0.269   0.246   0.004   0.750
E D  1T09A                347    0.507  73.073  -1.667   0.053   0.005   0.942
E N  1T09A                348    0.577  84.458  -1.668   0.053   0.005   0.942
E N  1T09A                349    0.395  57.769  -0.654   0.246   0.004   0.750
E K  1T09A                350    0.565 116.138  -0.392   0.502   0.002   0.495
E E  1T09A                351    0.459  80.222   0.992   0.522   0.016   0.462
B L  1T09A                352    0.045   8.203   0.638   0.858   0.002   0.139
E A  1T09A                353    0.312  34.404   1.151   0.923   0.002   0.076
E F  1T09A                354    0.558 111.930   1.620   0.970   0.001   0.030
B F  1T09A                355    0.052  10.436   0.677   0.970   0.001   0.030
B A  1T09A                356    0.017   1.873   0.697   0.970   0.001   0.030
E N  1T09A                357    0.488  71.487   1.801   0.970   0.001   0.030
E A  1T09A                358    0.226  24.949   2.342   0.970   0.001   0.030
B L  1T09A                359    0.015   2.746   1.738   0.988   0.000   0.012
B E  1T09A                360    0.148  25.908   1.126   0.988   0.000   0.012
E E  1T09A                361    0.477  83.384   2.290   0.988   0.000   0.012
B V  1T09A                362    0.027   4.119   0.994   0.988   0.000   0.012
B S  1T09A                363    0.017   2.016   1.481   0.988   0.000   0.012
E I  1T09A                364    0.346  64.010   1.851   0.988   0.000   0.012
E E  1T09A                365    0.434  75.907   2.319   0.988   0.000   0.012
B T  1T09A                366    0.030   4.119   0.678   0.970   0.001   0.030
B I  1T09A                367    0.112  20.794   0.975   0.923   0.002   0.076
E E  1T09A                368    0.688 120.246   1.428   0.858   0.002   0.139
E A  1T09A                369    0.694  76.490   1.248   0.246   0.004   0.750
E G  1T09A                370    0.501  39.413  -0.453   0.018   0.019   0.964
E F  1T09A                371    0.316  63.321   0.857   0.018   0.047   0.935
B M  1T09A                372    0.253  50.605   1.242   0.021   0.279   0.699
B T  1T09A                373    0.087  12.109   0.800   0.064   0.216   0.721
E K  1T09A                374    0.350  71.913   0.409   0.199   0.152   0.649
B D  1T09A                375    0.118  17.004  -2.436   0.278   0.093   0.628
B L  1T09A                376    0.058  10.565  -0.085   0.278   0.093   0.628
B A  1T09A                377    0.136  15.009  -1.432   0.268   0.043   0.689
B A  1T09A                378    0.273  30.041  -1.067   0.184   0.043   0.773
E C  1T09A                379    0.397  55.795  -0.834   0.191   0.086   0.723
B I  1T09A                380    0.196  36.353  -1.108   0.184   0.043   0.773
E K  1T09A                381    0.540 111.057  -0.851   0.191   0.086   0.723
E G  1T09A                382    0.462  36.367  -1.574   0.191   0.086   0.723
E L  1T09A                383    0.359  65.641  -1.302   0.184   0.043   0.773
E P  1T09A                384    0.509  72.156  -1.274   0.191   0.086   0.723
E N  1T09A                385    0.497  72.819  -0.990   0.191   0.086   0.723
E V  1T09A                386    0.395  60.681  -1.334   0.184   0.043   0.773
E Q  1T09A                387    0.616 109.946  -1.287   0.113   0.043   0.844
E R  1T09A                388    0.575 131.698  -1.296   0.058   0.017   0.925
E S  1T09A                389    0.416  48.779  -1.376   0.053   0.043   0.903
E D  1T09A                390    0.457  65.911  -0.372   0.052   0.084   0.864
E Y  1T09A                391    0.337  72.038  -0.397   0.019   0.141   0.840
B L  1T09A                392    0.114  20.855  -0.228   0.019   0.141   0.840
B N  1T09A                393    0.166  24.244  -0.038   0.053   0.043   0.903
B T  1T09A                394    0.023   3.259   0.450   0.879   0.010   0.111
E F  1T09A                395    0.358  71.871   0.956   0.970   0.001   0.030
E E  1T09A                396    0.310  54.209   1.692   0.970   0.001   0.030
B F  1T09A                397    0.033   6.663   0.646   0.970   0.001   0.030
B M  1T09A                398    0.026   5.263   0.786   0.970   0.001   0.030
E D  1T09A                399    0.432  62.251   1.712   0.988   0.000   0.012
B K  1T09A                400    0.222  45.645   2.045   0.988   0.000   0.012
B L  1T09A                401    0.019   3.552   1.482   0.988   0.000   0.012
B G  1T09A                402    0.079   6.178   1.295   0.970   0.001   0.030
E E  1T09A                403    0.563  98.321   2.190   0.970   0.001   0.030
E N  1T09A                404    0.262  38.371   2.370   0.970   0.001   0.030
B L  1T09A                405    0.026   4.816   1.107   0.970   0.001   0.030
E K  1T09A                406    0.431  88.595   2.001   0.970   0.001   0.030
E I  1T09A                407    0.625 115.570   1.848   0.970   0.001   0.030
E K  1T09A                408    0.326  67.120   1.608   0.923   0.002   0.076
B L  1T09A                409    0.166  30.450  -0.486   0.858   0.002   0.139
E A  1T09A                410    0.553  60.996   0.718   0.782   0.003   0.216
E Q  1T09A                411    0.709 126.681   1.193   0.694   0.003   0.303
E A  1T09A                412    0.464  51.155   0.085   0.246   0.004   0.750
E K  1T09A                413    0.625 128.604   0.563   0.109   0.005   0.886
E L  1T09A                414    0.652 119.308  -0.767   0.003   0.003   0.994

Explain the output.  Go back.
```

---
